# Development and deployment of a functional 3D-bioprinted blood vessel

**DOI:** 10.1038/s41598-025-93276-y

**Published:** 2025-04-05

**Authors:** Annika C. Dell, Jamie Maresca, Bruce A. Davis, Toshihiko Isaji, Alan Dardik, John P. Geibel

**Affiliations:** 1 The John B. Pierce Laboratory, Inc, New Haven, CT 06519 USA; 2 Fraunhofer IMTE, Fraunhofer Research Institution for Individualized and Cell-Based Medical Engineering, 23562 Lübeck, Germany; 3 Department of Cellular and Molecular Physiology, Yale School of Medicine, New Haven, Connecticut, CT 06510 USA; 4 Division of Vascular Surgery, Department of Surgery, Yale School of Medicine, New Haven, Connecticut, CT 06510 USA

**Keywords:** Cardiovascular models, Biomedical engineering, Biomaterials - cells, Tissues

## Abstract

Creating vascular structures in vitro via bioprinting to replace damaged or missing vasculature has significant advantages over current surgical methods of vessel replacement. Using rat fibroblasts and smooth muscle cells, we have bioprinted a rat aorta using a rotating mandrel method to create the tubular replacement structure. Then, the 3D-bioprinted aortas were implanted into rats to determine their functionality long-term in vivo. The implanted vascular conduits were well-tolerated, well-incorporated into native vasculature, and showed the physiological behavior of a native vessel. The development and deployment of 3D-bioprinted vessels for repair of large vessels in an animal model paves the way for advancements in the treatment of vascular disease in humans.

**Supplementary Information:**

The online version contains supplementary material available at 10.1038/s41598-025-93276-y.

Bioprinted tissues are three-dimensionally (3D) engineered, functional tissues using living cells and biomaterials^1^. When bioprinting tissue, the aim is to accurately replicate the three-dimensional architecture, along with the physical and biochemical environment of the native tissues, allowing for functional tissue for in vivo or in vitro use^2^. This can be achieved through various printing modalities and methods, and has found numerous biomedical applications, including in vitro disease modeling, drug testing, and regenerative medicine. Stemming from the motivation to work towards bioprinting organ structures, one important element of tissue engineering is vascularization, as vascular tissues, or blood vessels, provide nutrient transport and the disposal of cell waste products in tissue. If there is no vasculature in tissue structures, the size and complexity of an engineered tissue is limited, as the lack of nutrition and accumulation of waste will inevitably lead to cell death in bioprinted tissue structures. Additionally, there are numerous pathologies that would benefit from the ability to replace or repair damaged vessels using vasculature that has been manufactured in vitro using a bioprinter. 

For example, cardiovascular disease (CVD) remains one of the most prevalent causes of death worldwide^3^. The growing numbers of people afflicted with a vascular disease, coupled with the high mortality and morbidity posed by these diseases, is a significant and growing current challenge in medicine^4,5^. Common forms of CVD include coronary artery disease and peripheral arterial disease (PAD), which are commonly characterized by atherosclerosis, which is defined as the buildup of plaque in the vessel lumen, resulting in stiffening of the arterial wall. This can cause stenosis, leading to ischemic injury that can result in patients needing vessel replacement^6,7^. In some extreme cases, particularly among diabetic PAD patients, foot or leg amputations may be necessary due to poor circulation. Currently, vascular grafts sourced from the patient (autologous grafts), or synthetic grafts are used to replace or circumvent the damaged vessel. However, autologous grafts are limited by availability, donor site morbidity, the need for invasive surgery, and have an approximately 30% 10-year failure rate^8^. Synthetic grafts, such as expanded polytetrafluoroethylene (Gortex) or polyethylene terephthalate (Dacron) are limited to large-diameter blood vessels (> 6 mm), due to thrombus formation and frequent structural failure in small-diameter grafts^9^. In addition, synthetic grafts have low patency rates, meaning that they begin to leak over time. Additionally, they can be prone to biofilm growth, which requires further surgery to remove^10,11^. Other specialties beyond endovascular and cardiovascular applications also require vascular structures. For example, in severe cases of chronic kidney disease (CKD), a vascular access (VA) can be needed to perform efficient hemodialysis. The native arteriovenous fistula (AVF) is an autogenous option, which makes use of the patient’s own vasculature to create a vascular access site for the dialysis patients require on a regular basis^12^. However, only 60% of all AVFs are functional for dialysis after twelve months^13^. Thus, a small-diameter vessel replacement option would be beneficial in a variety of medical specialties. 

Current clinical treatment strategies of vascular pathologies, as described above, can fail for several reasons. Tissue engineering approaches, of which there are numerous, provide a solution for the biofabrication of patient-specific, biological vascular structures. Several tissue engineering methods of producing vascular conduits have been employed, differing in the support mechanism to give the tubular shape structure, material and cell type used, and construction method. Often, blood vessels are created by using a scaffold, a three-dimensional porous matrix onto which cells may be loaded. The scaffold provides a surface for cells to adhere, proliferate, and generate the extracellular matrix (ECM)^14^. These tissue engineering approaches can generally be categorized into: utilizing scaffolds comprised of polymers or biomaterials (degradable or nondegradable)^15,16^, decellularization processes, cell-sheet methods, and molding techniques^17^. 

The classic tissue engineering method centers around using bioreactors to mature scaffolds loaded with cells: depositing cells in a synthetic or natural scaffold, which allows for the orientation and differentiation of cells into three-dimensional tissue^18,19^. The developing tissue can then be further cultured in a bioreactor to allow for maturation and development of normal physiological functions^18,20^. One of downsides of using bioreactors is that their use is time-consuming, often taking weeks for a tissue-like structure to become viable^15,21^. 

Native arteries are composed of three layers: (1) the intima, the innermost layer, containing endothelial cells, (2) the lamina, an elastic layer containing vascular smooth muscle cells (SMCs) as well as layers of collagens I & II and elastin lamellae, and (3) the adventitia, containing primarily fibroblasts. Weinberg and Bell were pioneers in tissue engineering, developing a vessel structure by casting collagen with smooth muscle cells (SMCs) and fibroblasts (FCs), using a glass mandrel to create the blood vessel lumen rather than a degradable scaffold^22^. In 1998, L’Hereux et al. fabricated tubular tissue via autologous cell-derived ECM sheets—a cell sheet assembly method—to create a tri-layered vessel structure, also containing SMCs and FCs. These cell sheets are wrapped around a mandrel, thereby giving them their vessel-like shape^23,24^. These methods are generally referred to as “scaffold-free” methods, as the support structure is not a sacrificial biomaterial or polymer, but rather a mandrel, often made of stainless steel or glass. The scaffold-free method combats some of the limitations faced with the use of artificial scaffolds, as described above. Scaffold-free constructs allow for a higher biocompatibility and cell density with a low chance of a foreign body response, as the inherent capacity of cells to secrete an ECM, which acts as a support structure for the cells, is exploited^25,26^. Additionally, extruded cells deposited on an artificial scaffold need time to grow into a vascular structure—this process can take weeks or months of culturing time^16^. If using a high cell concentration and using scaffold-free techniques to create the hollow vascular structure, excessively long culturing times following bioprinting can be omitted. 

Tissue-engineered vascular structures can elicit an immune response upon implantation, including precocious reabsorption, fibrosis of the implant and/or rejection of the implant, thereby leading to failure of the intervention^27^. Additionally, the simultaneous degradation process of the scaffold (by enzymatic processes or hydrolysis) and neo-tissue formation must be carefully coupled. If, for example, the degradation rate of the scaffold exceeds the rate of tissue formation, the scaffold could prematurely be absorbed in vitro, destroying the structure required by cells to develop new tissue. In decellularization methods, complexities arise in the process of the decellularization itself – often, treatment of the scaffold to remove antigens that are detrimental to the scaffold is necessary, in addition to the selection of the most fitting detergent for the tissue^28^. The described difficulties posed by artificial scaffolds of various types may be circumvented by using biopolymers that mimic the native structural proteins in the ECM, in combination with a scaffold-free method. The next step in tissue engineering is the continued use and improvement of using bioprinters, which allow for controlled placement of cells suspended in a bioink, rather than the less precise method of seeding cells and allowing them to mature in a scaffold. 

3D bioprinting is an emerging technology that allows for the creation of complex cell-laden structures. Our group took advantage of a scaffold-free bioprinting approach for creating cylindrical blood vessels. Other groups have also employed scaffold-free methods in bioprinting vasculature. For example, Norotte et al. employed the use of cylindrical building blocks, printing cellularized cylinders concomitantly with agarose rods, that were effectively used as mandrels^29^. The ‘Kenzan’ method, now commercialized in a system named Regenova, commercialized in Japan by Cyfuse Biomedical, K.K., and in US by Amuza, Inc., makes use of 160 micrometer thick stainless-steel microneedles (“kenzans”) to provide spatial organization, rather than a hydrogel^30^. Cell spheroids are first assembled and are then speared onto the “kenzans” or micro-needles using a robotic arm^31^. Gao et al. used a rotating stainless-steel rod to fabricate vascular constructs containing micro- and macro-channels^32^. Some groups use scaffold-free molding approaches to create the hollow cylindrical structure that is to become a blood vessel. For example, Swartz et al. make use of a mold to handmake their fibrin-based vessel structure. However, this approach does not allow for the creation of all layers present within the vessel walls (tunica intima, tunica media, tunica adventitia) – only the SMCs are used to create a tunica media, and a thin layer of endothelial cells if created along the inner lumen^33^. The bioink used in this work contains smooth muscle cells (SMC) and fibroblasts (FC) – the elastic smooth muscle cell and fibroblast mixture layer mimics the tunica media, and the layer of fibroblasts mimics the tunica adventitia. In our method, no endothelial cells were used to imitate the tunica intima, as we expected self-endothelialization by progenitor cells present in the blood. Additionally, a hydrogel kit containing hyaluronic acid, gelatin, and polyethylene glycol diacrylate (PEGDA) is used, as this constellation of hydrogels provides compression strength, lubrication, and hydration, while allowing for cell motility, adhesion, and proliferation.

## Materials and methods

### Bioink preparation

To construct the vascular conduits, rat venous SMCs and rat aortic FCs were grown in individual cell culture flasks and harvested at passage 10 or less upon achieving between 80% and 90% confluence. A hydrogel-based bioink was used in the Organovo printer to generate a single vascular conduit. The HyStem-C Kit by Advanced BioMatrix (BICO Group AB, Gothenburg, Sweden) used contains hyaluronic acid, gelatin, and polyethylene glycol diacrylate (PEGDA). The bioink was made with the harvested SMCs and FCs by encapsulating the cells at a density of 100 × 10^6^/mL in the crosslinked hydrogel mixture. Two sets of bioink were generated, differing in cell types but otherwise remaining the same: the first being comprised of 70% SMCs (42 × 10^6^ cells), 30% FCs (18 × 10^6^ cells), and the hydrogel mixture, while the second consisted of 100% FCs (60 × 10^6^ cells) and the hydrogel mixture. Cells and hydrogel were combined, and a homogenous mixture was achieved by gently drawing the mixture up and down in an uncapped 0.5 ml Hamilton syringe before the hydrogel has cured. Then, the bioink is drawn into the uncapped Hamilton syringe, capped with a rubber cap, and allowed to chill and cure on ice. This is the bioink.

### Bioprinting protocol

**Fig. 1:**
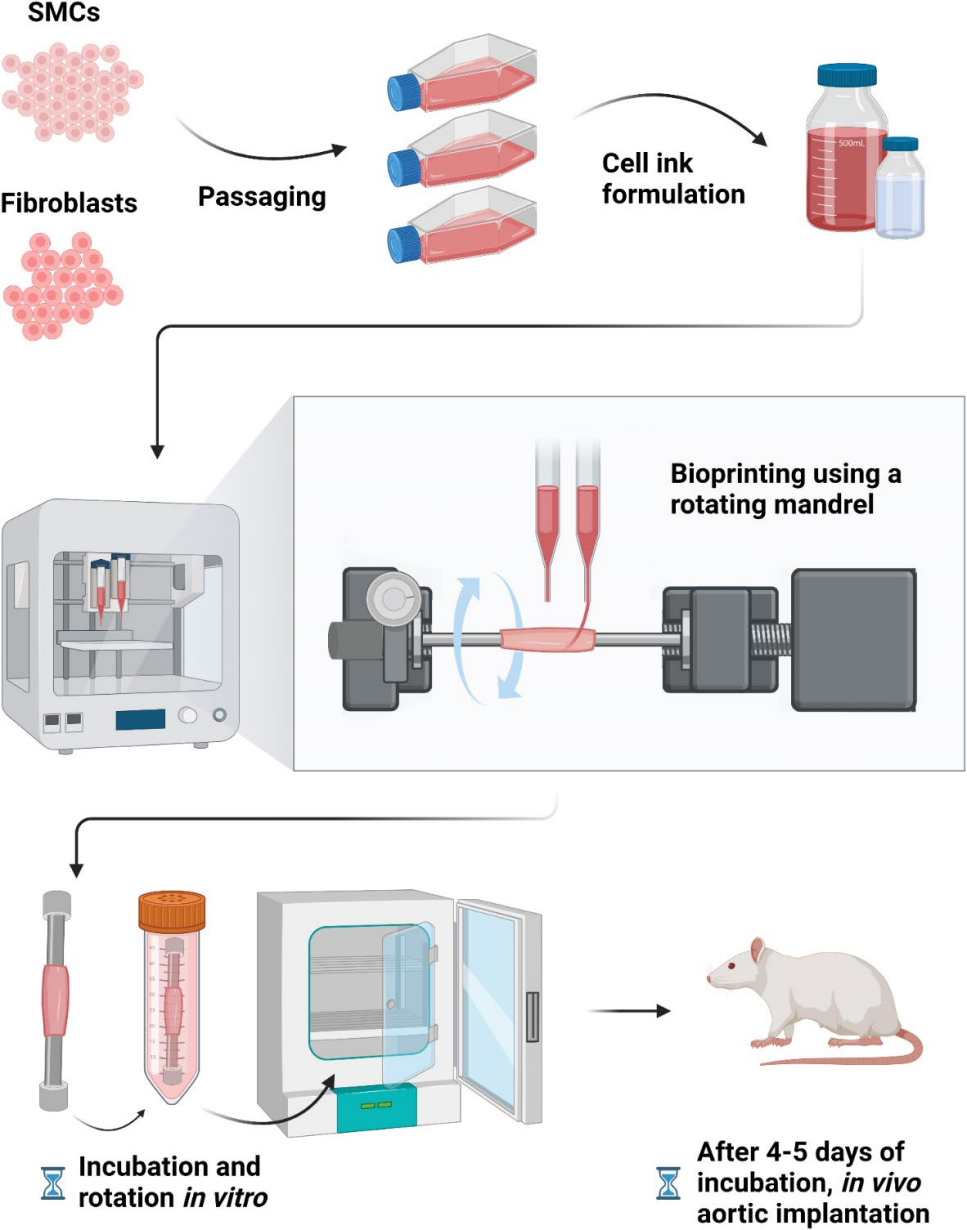
Bioprinting process using the Organovo bioprinter and printing setup to allow for bioprinting onto a rotating mandrel.

The chilled 0.5 ml Hamilton syringes containing the bioink are loaded into the printer. The bioprinter (NovoGen MMX Bioprinter™; Organovo, Inc., San Diego, CA) uses a mechanical extrusion mechanism to deposit the bioink onto a surface, allowing the fabrication of three-dimensional tissue structures. In the case of a conventional bioprinting setup, the extruded bioink would be extruded onto the print bed, a flat surface. In our setup, the bioink extruded from the syringe tip was deposited on a rotating stainless steel rod, a mandrel (diameter = 3 mm), driven by a small motor (see Fig. S1 in Supplementary Materials). As the printhead moves along the length of the mandrel, the mandrel rotates, so that a cylindrical structure is achieved in ~ 6 min. A schematic of the printing process is shown in Fig. 1.

Two layers of the 70:30 SMC:FC and one layer for the FC outer layer were printed on the 3 mm rotating mandrel, resulting in a 3D-bioprinted vascular conduit with a length of 10 mm, comprised of three layers each 500 μm thick, with the first two inner layers consisting of SMC and FC the outer layer containing FC only, thereby emulating the layers found in blood vessel walls.

### Culture condition

in vitro. Post-printing, the mandrel is removed from the motor and rubber bumpers are placed on the proximal and distal ends of the rod to protect the conduit from damage in the culture tube.

The prints were placed in a Dulbecco’s Modified Eagle’s Medium (DMEM)-based growth medium containing fetal bovine serum, fibroblast growth factor, epidermal growth factor, transforming growth factor beta, insulin, hydrocortisone, l-glutamine, vitamin C and copper.

The mandrel with the vessel conduit still attached was placed into a 50 cc Falcon tissue culture tube (vented cap) with 20–25 ml of cell medium and was incubated 30 °C incubator for 24 h. The print is then transferred to a 37 °C incubator for 24 h. The media is replaced and the tube containing the mandrel and print are placed into a spinning incubator at 37 °C for an additional 24–48 h. The print is removed from the stainless-steel rod and was typically implanted within 24 h. If left in the incubator too long, the print becomes more difficult to remove from the mandrel.

in vivo. To study the in vivo behavior of bioprinted vasculature, animals were divided into a control group (*n* = 20) and an experimental group (*n* = 20). The experimental group received aortic conduits within 24 h following completing the in vitro incubation. Male Sprague-Dawley rats, weighing 210–275 g, (Charles River Wilmington, USA) were used. The animals were anesthetized with IsoThesia™ 99.9%/mL. A midline laparotomy was performed to gain access to the aorta. The aorta was cross-clamped and an arteriotomy was performed to explant a 10 mm segment of the infrarenal aorta. The vascular conduit was implanted in the aorta via an end-to-end arterial anastomosis using 2 − 0 silk sutures. Incisions were closed with 4 − 0 silk sutures and the operation site was irrigated with saline solution. Animals fasted overnight after the procedure, and thereafter resumed a normal soft feed diet while being monitored daily for fluid intake, stool output, and weight gain. Animals were euthanized at post-operative day 7, 30, and 60 (Groups C1/E1, C2/E2, C3/E3, respectively) via an over-inhalation of isofluorane. The vessel segment was then explanted. The twenty rats in the control group were treated just as those in the experimental group were: a midline laparotomy was performed to expose the aorta, and the aorta was cross-clamped for the same amount of time that a procedure would take. Then, the clamps were released and the incision was closed – no implantation or explantation of vascular structures was performed in the control animals. Animal handling and care was approved and performed according to the Yale Animal Care and Use Committee guidelines [Protocol ID #2016‐10896]. All efforts were made to minimize suffering following institutional guidelines.

**Fig. 2:**
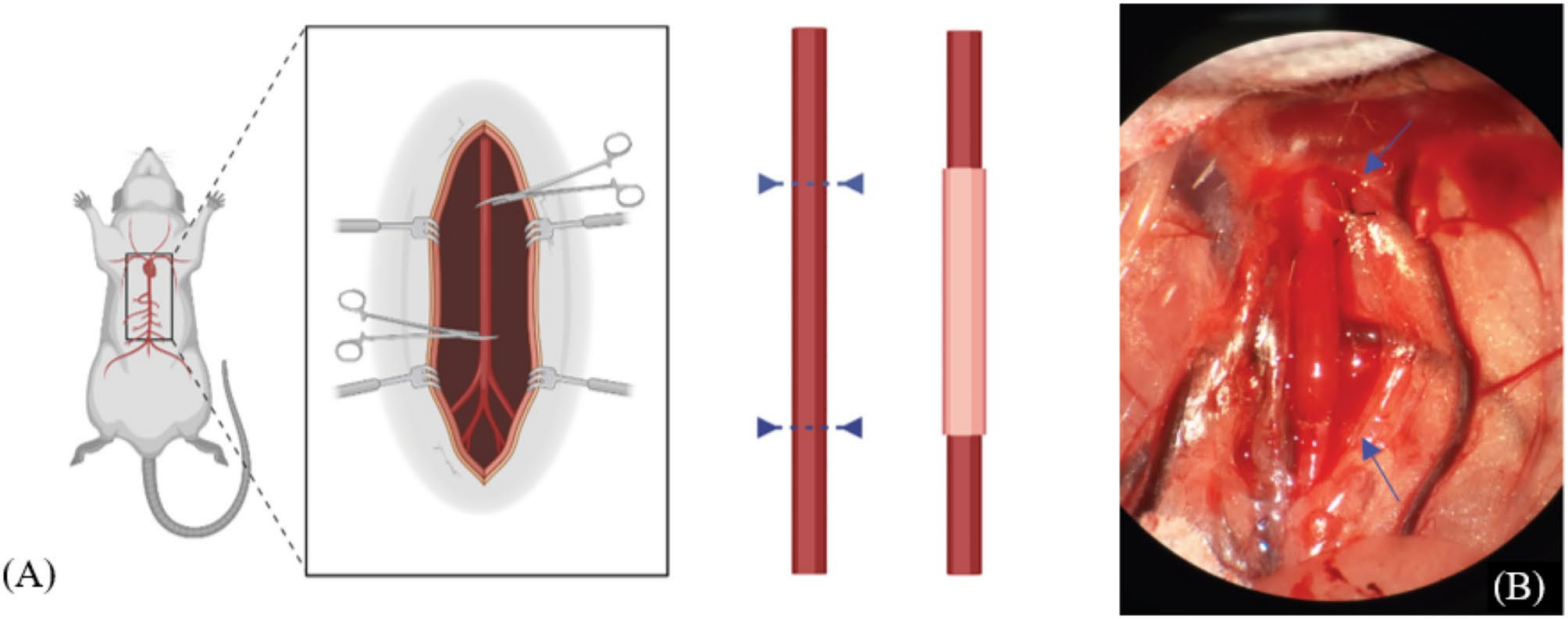
(**A**) Shows a schematic of the implantation method (**B**) An implanted aorta in situ immediately after implantation.

## Results

Figure 2 shows the implantation method and an image of an implanted aorta *in situ*. The bioprinted vascular conduits remain patent after printing and have a tensile and mechanical strength similar to native blood vessels, though further evaluation is required to prove the mechanical behavior^9^. Patency of the implanted conduits is proven through animal survival, as the lack thereof would quickly lead to significant symptoms or death. In both the control and experimental group, neither aneurysmal behavior nor bleeding at the suture site were observed, and no animals developed post-surgical complications. The visible inflammation reaction was minimal – vascular conduits were incorporated well. The 10–20 mm aortic replacements can withstand pressure and trauma of implantation and can survive in vivo as aorta replacements. No anticoagulation or anti-platelet drugs were given. Normal animal mobility and 10% weight gain per week were observed in both the experimental group and control group following surgery, which is a normal result, proving general animal health. Table 1 shows the results of the implantations into the experimental group: a high viability post operation was observed post-operation. Only one rat did not survive the surgical procedure, due to ischaemia caused by cross-clamping. Thus, it appears that the lack of toxicity associated with the degradation of a biological scaffold renders these vascular conduits suitable for implantation in vivo^9^. All rats in the control group survived the experiment.

**Table 1:** Experimental (E) and control group (C) results: animal survival rates post-implantation of our bioprinted aorta for each of the three timeframes observed: 7, 30, and 60 days.

Trial group	Number of rats rmplanted	Days post-implantation	Number of rats survived	Percent survival
E1	5	7	4	80%
E2	5	30	5	100%
E3	10	60	10	100%
C1	5	7	5	100%
C2	5	30	5	100%
C3	10	60	10	100%

## Discussion

Our method of combining extrusion bioprinting with a rotating mandrel allows for an uncomplicated means of creating a viable cylindrical vascular conduit. Using this method, mechanically and biologically functional blood vessels of different lengths and diameters can be fabricated. The limitations of this method are the number of cells in large or long vessel structures and the current inability to print bifurcations. Large-diameter blood vessels, such as a human aorta or iliac arteries, have not been tested by this group thus far, but would prove to be complicated purely through the sheer volume of cells and hydrogel necessary for such a feat. In addition, venous structures would require an added layer of complexity due to the construction of valves and a thin wall that could prove to be brittle. Hence, our work is focused on arterial vascular structures.

We have demonstrated that we can circumvent the problems posed by biomaterials-based support structures, including immunogenicity, degradation rate, toxicity of degradation products, host inflammatory responses, fibrous tissue formation due to scaffold degradation, and mechanical difference to the native issues^34^. These issues inhibit or completely prevent implantation and long-term functionality and interfere with the biological function of the bioprinted tissue structure. The collagen present in the hydrogel used in our bioink is a major component of the ECM in blood vessels that helps limit high strain deformation on the blood vessel walls^35^. This helps create a structurally stable bioprinted blood vessel that is geometrically and structurally similar to native blood vessels. Additionally, collagen is ideal in blood vessel engineering due to its low inflammatory and antigenic response. The stainless-steel mandrel used in our work in no way alters cell behavior or proliferation and does not interfere with the completed vessel, unlike other scaffold-based methods. The vascular conduit has a smooth surface, indiscernible from a native blood vessel, and can be removed from the mandrel post- in vitro cultivation with ease. The mandrel method allows easier handling than a micro-needle method, for example, and allows for an uncomplicated means of adding multiple layers and thereby emulating the three layers of a blood vessel, unlike molding approaches. In addition, our vascular conduit was achieved without using a bioreactor or decellularization processes, thus shortening the timespan needed. Hence, our method is a viable means of quickly creating a vessel, as the use of bioreactors can increase the time needed to create such a vessel dramatically by days or weeks^21,36^. Furthermore, eschewing the bioreactor allows for simplifying the process of developing the vessel conduits, as a customized bioreactor is not necessary.

We have shown that bioprinted vascular conduits using rat smooth muscle cells (SMCs) and rat fibroblasts (FCs) remain stable in vivo in timeframes spanning from 7 to 60 days. To our knowledge, this is the longest amount of time that a multilayer biological blood vessel conduit, that has been bioprinted using a scaffold-free method, has successfully remained implanted into an animal model. Quint et al. describe a decellularized tissue-engineered vessel (TEV) made from human vascular smooth muscle cells and subsequent implantation into a rat model^37^. This group’s process, involving isolating smooth muscle cells from explanted patient tissue and developing the TEV using a bioreactor, takes several weeks. Additionally, this group implanted these TEVs into rats, which showed a high patency rate, no evidence of graft dilatation, and neotissue formation. Itoh et al. describe using a (scaffold-free) micro-needle method to create a vascular structure in around eight days^31^. These structures were then implanted, observed, and explanted after only five days. Jang et al. describe using an extrusion bioprinter to print mesenchymal stem cells onto a steel mandrel. This group implanted their vessels into canines over the course of two weeks but were confronted with occlusions in the printed implanted vessels^38^.

Based on our results, we believe that our method can easily be applied to other cell types, specifically human cell types, and thus could be used in numerous clinical applications—from implantation to in vitro testing. Though this work describes rat vasculature,our method produces results that are not miniaturized, e.g., the size of our vascular conduit is comparable to that of a small human artery^39^. Thus, little stands in the way of developing a human artery with regards to methodology, construction time, and geometry. Pathologies such as diabetic foot and arteriosclerosis could be treated, and arteriovenous fistulas (AVF) for use in hemodialysis patients could be manufactured, avoiding the problems caused by synthetic or autologous vasculature. Additionally, our method has potential for pediatric patients, as implanted synthetic vessel structures do not grow with patients^40^. Furthermore, due to the relative speed of our method, individualized vessel structures can be developed by using patient cells to print the vascular conduit, albeit some time must be invested into isolating and passaging patient cells. The resulting individualized vessel would circumvent issues mentioned thus far, including potential rejection of the implant as foreign tissue^27^. However, further investigation to evaluate this bioprinted vascular conduit, such as characterizing the mechanical properties, proving neointima formation and cellular arrangement, as well as performing hematoxylin and eosin (H&E) staining and immunohistochemistry, should be done before implantation into a patient can be considered.

It can be concluded that our bioprinted vascular conduits are biologically and mechanically functional vessels with a complex wall composition, proven to be usable in long-term implantation in an animal model^41^. Our vascular conduits demonstrated full patency after three to four days of incubation and were quickly suitable for implantation, with printing time being less than ten minutes^42^. We believe that our method could be translated to a clinical setting, after isolating the necessary cells from patients, thereby quickly developing an individualized, small-diameter blood vessel for a number of applications. These results pave the way for the use of bioprinted vessels in humans and in furthering the development of bioprinted tissue structures.

## Electronic supplementary material

Below is the link to the electronic supplementary material.

Supplementary Material 1

Supplementary Material 2

Supplementary Material 3

## Data Availability

The raw data supporting the conclusions of this article will be made available by the corresponding author, without undue reservation, to any qualified researcher.
