# Supplementary material for: Development and deployment of a functional 3D-bioprinted blood vessel
Source: Sci Rep. 2025 Apr 5;15:11668. doi: 10.1038/s41598-025-93276-y (PMC11972400; doi:10.1038/s41598-025-93276-y)
Supplement: Supplementary file 1 — Supplementary Material 1 [file 41598_2025_93276_MOESM1_ESM.docx]

Supplementary Materials for

Development and Deployment of a Functional 3D-Bioprinted Blood Vessel

A. C. Dell, J. Maresca, B. A. Davis, T. Isaji, A. Dardik, J. P. Geibel

Corresponding authors: [Annika.dell@imte.fraunhofer.de](mailto:Annika.dell@imte.fraunhofer.de), [john.geibel@yale.edu](mailto:john.geibel@yale.edu)

**The PDF file includes:**

Figure S1


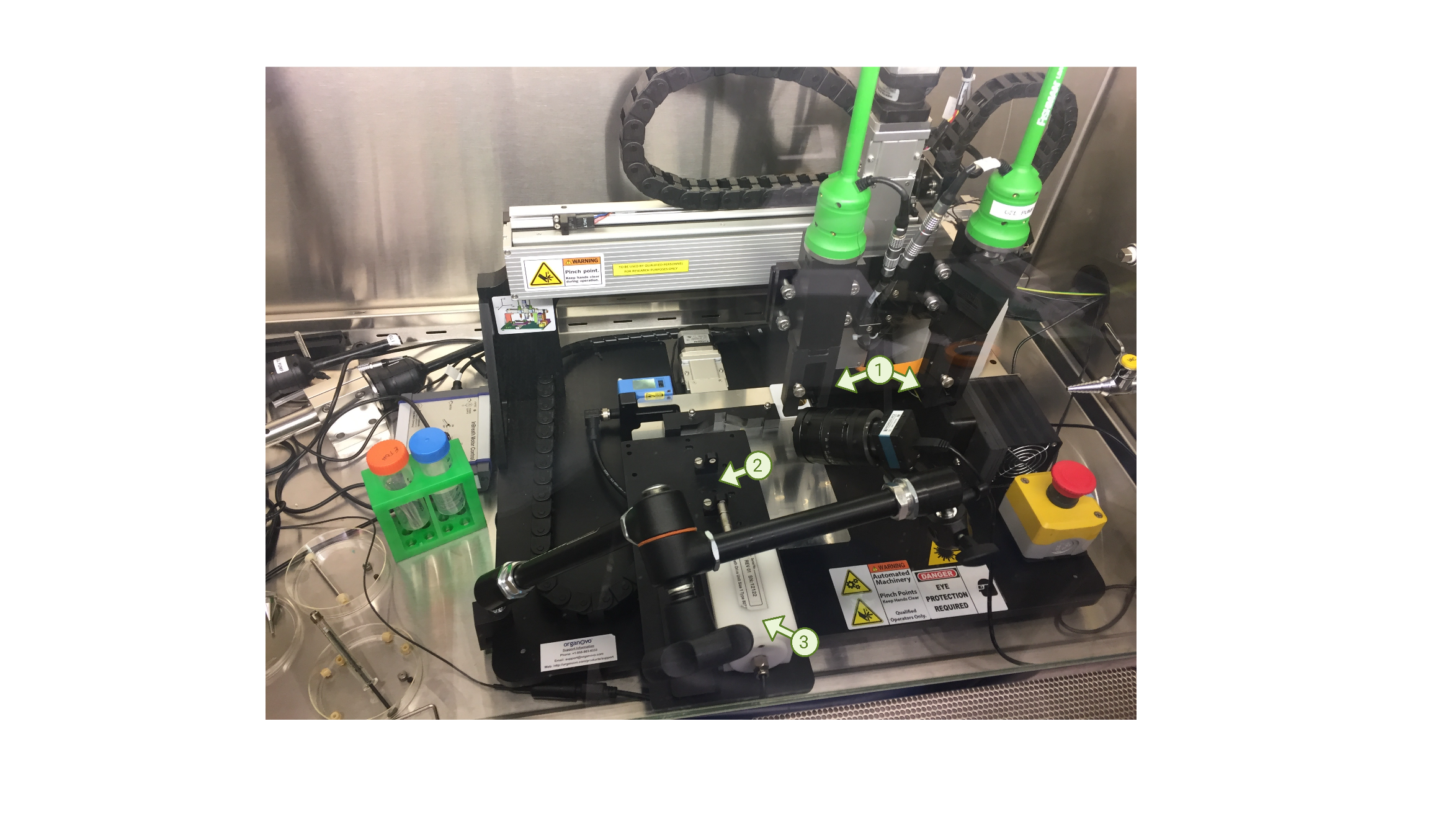


**Fig S1.:** Organovo NovoGen MMX Bioprinter. **1.** Printheads: Hamilton syringes with attached needle are inserted into each printhead chamber. **2.** The mandrel is inserted into holders, which are in turn connected to the motor (3.), which rotates the mandrel. **3.** Motor responsible for rotating the mandrel.
